# 

**Published:** 1916-06-03

**Authors:** 


### Provision for Disabled Soldiers at Cardiff.

At the monthly meeting of the board of management of King Edward VII.'s Hospital, Cardiff, it was decided, on the proposition of Colonel Bruce Vaughan, to provide accommodation for six disabled soldiers in one of the houses belonging to and adjacent to the hospitol. The idea is to make provision in Cardiff for men belonging to the neighbourhood who have been discharged from the Army on account of incurable or chonic injury, but who will continue to require hospital treatment.

At the same meeting further gifts towards the proposed extensions were announced, including 1,000 guineas each from Messrs. Insoles, Ltd., and Mr. Charles Radcliffe. And these generous gifts, with the recent history of the Cardiff Hospital confirms its past experience, that the more ready it is to extend its usefulness, the more readily it finds the public is prepared to back its courage by increasing support. It is gratifying to be able to record that within comparatively recent months funds amounting to no less than £40,000 have already been subscribed, either for the endowment of beds or towards the proposed extensions.

The Royal Commission on University Education in Wales are timed to pay a visit to Cardiff during the week commencing June 19, when they will find in King Edward VII.'s Hospital an institution which will fulfil, in an ideal manner, the requirements of a medical school, whilst a medical school will, in its turn, add greatly to the usefulness and efficiency of the hospital.

### HOSPITAL RESULTS IN 1915.

**Charing Cross Hospital.**—The annual report for 1915, which for motives of economy is not being so widely distributed as the issues of previous years, is a volume of 327 pages, 260 of which are required for the record of present and past benefactors. The main features of the report of the council are contained in our report of the annual general court of governors (The Hospital, April 1, page 19). The progress in the work of money-raising reflects great credit on those concerned; the following records are very striking:—Subscriptions: 1913, £2,822; 1914, £4,331; 1915, £9,512. Donations: 1913, £4,878; 1914, £9,582; 1915, £20,488.

**Hospital for Sick Children, Great Ormond Street.**—The annual report takes the customary neat form, and is now printed on thin paper, which must make a welcome difference in cost of postage. The average cost per in-patient has been reduced by slightly over one shilling. There was a decrease of £5,063 on the total income for 1915. The secretary, Mr. E. Stewart Johnson, who is on active service, has for family reasons changed his name to E. Stewart Darmady.

**Poplar Hospital for Accidents.**—The report is for the diamond jubilee year of the hospital, which must be a very happy institution, producing as it does year after year its well-arranged and business-like record of twelve months of excellent work carried out without getting into debt. The manner in which the affairs of the charity are conducted is well rewarded by many business men and the City Companies, who are never too busy to notice the way in which their contributions are recorded and expended. The income for 1915, £19,858, was the second largest ever received in the history of the charity. A meeting at "The Baltic" took the place of the usual festival dinner, and induced a collection of £7,000, more than half of which sum was gathered together by the officials of the Port of London Authority.

**Manchester Children's Hospital.**—This hospital has no accountancy difficulty in separating its in-patient and out-patient expenditure, as the hospital and out-patient department are in separate districts. Owing to the shortage of beds in other hospitals caused by the reception of military patients, extra pressure on the accommodation of this children's hospital has been noticeable, and only serious and urgent cases were taken. Competitive tenders for food supplies have been considered and accepted monthly. The total income was £9,301, and the total expenditure £12,305. The method of presenting accounts is slightly behind the times.

**St. Thomas's Hospital.**—It is claimed that this great hospital is carrying on its civil work and answering all the calls made upon it, and is serving also as one of the great war hospitals of the kingdom, having under its control the 5th London General Hospital, which has 530 beds in the hospital and the specially erected adjoining huts. On the civilian side the total number of in-patients treated has been very nearly up to that in previous years, and that of new out-patients has fallen from 69,895 to 62,544. The total income was £96,773 and the expenditure £94,418. Under "Surgery and Dispensary" strict economy has resulted in a saving of nearly £630, but under most other headings there has been an increase, particularly heavy in the instance of "Provisions," chiefly caused by the larger number of persons to feed. The report of the Samaritan Fund is dealt with on page 205, under "Hospital and Institutional News."

### PASSING EVENTS AND TOPICS.

### Mr. C. W. Thies.

The Council of the Royal Eye Hospital, Southwark, at their meeting held on May 10, unanimously adopted a handsome resolution of thanks to Mr. Conrad W. Thies for the services rendered by him during the six months he has acted as honorary secretary to the hospital.

### The Edith Cavell Hospital in France.

A French committee is in the course of formation for the organisation in France of the Edith Cavell Hospital and College of Nursing, where it is proposed to train educated French women for the nursing profession. Mrs. Bedford Fenwick has been invited to join the committee and to act as its nursing adviser.

### Royal College of Surgeons of England.

The subjects and conditions of forthcoming prizes to be awarded by the College of Surgeons have been announced. The Triennial Prize, the dissertations for which have to be delivered before December 31, 1918, takes the form of the John Hunter Medal in gold, to the value of 50 guineas (or a bronze medal and an honorarium of £50). The subject is "The Development of the Hip-Joint and the Knee-Joint of Man." The subjects for the Jacksonian Prize for 1916 and 1917 respectively are: "Methods and Results of Transplantation of Bone in the Repair of Defects Caused by Injury or Disease" and "The Causation, Diagnosis, and Treatment of Traumatic Aneurysm, including Arterio-Venous Aneurysm." The dissertation for 1916 must be delivered before December 30, and that for 1917 before December 31, 1917. Candidates must be Fellows or Members of the College, not on the Council.

### Money Grants to Incurables.

At the annual meeting of subscribers of the Royal Society for Home Relief to Incurables, Edinburgh, it was stated that last year the expenditure exceeded the revenue by £751, and the Lord Provost of Edinburgh, in moving the adoption of the report, said it was hoped that notwithstanding the war claims and the extra burdens which had been thrown on the public generally, this society of 110 years' standing would receive due recognition and support from the public.

### The South London Hospital for Women.

Her Majesty the Queen has graciously consented to open, on July 4 next, the South London Hospital for Women, which has been built on a site facing Clapham Common. The hospital is officered entirely by women doctors, and has accommodation for eighty patients. The building will be opened free from debt, but the money for maintenance and upkeep has yet to be collected. The Marchioness of Londonderry is chairman of the board, and will be pleased to reply to any inquiries addressed to 88/90 Newington Causeway, S.E.

RATES OF SUBSCRIPTION: Twelve months, 6/6; Six months, 4/-; Three months, 2/-, post free to any address in the United Kingdom. Abroad, Twelve months, 8/8; Six months, 5/-; Three months, 2/6, post free.—Address The Subscription Dept., "THE HOSPITAL," The Hospital Building, 28/29 Southampton Street, Strand, London, W.C.

